# Midlife risk factors predict long-term hip fracture risk in women: a 35-yr follow-up

**DOI:** 10.1093/jbmrpl/ziag083

**Published:** 2026-05-08

**Authors:** Otto A Mustonen, Reijo Sund, Heikki Kröger, Joonas Sirola, Kenneth E S Poole, Toni Rikkonen

**Affiliations:** Kuopio Musculoskeletal Research Unit (KMRU), Institute of Clinical Medicine, School of Medicine, University of Eastern Finland (UEF), P.O. Box 1627 FI-70211, Kuopio, Finland; Kuopio Musculoskeletal Research Unit (KMRU), Institute of Clinical Medicine, School of Medicine, University of Eastern Finland (UEF), P.O. Box 1627 FI-70211, Kuopio, Finland; Kuopio Musculoskeletal Research Unit (KMRU), Institute of Clinical Medicine, School of Medicine, University of Eastern Finland (UEF), P.O. Box 1627 FI-70211, Kuopio, Finland; Department of Orthopedics, Traumatology and Hand Surgery, Kuopio University Hospital, Puijonlaaksontie 2, 70210 Kuopio, Finland; Kuopio Musculoskeletal Research Unit (KMRU), Institute of Clinical Medicine, School of Medicine, University of Eastern Finland (UEF), P.O. Box 1627 FI-70211, Kuopio, Finland; Department of Orthopedics, Traumatology and Hand Surgery, Kuopio University Hospital, Puijonlaaksontie 2, 70210 Kuopio, Finland; Department of Medicine, University of Cambridge, NIHR Cambridge Biomedical Research Centre, Addenbrooke’s Hospital, Hills Rd, Cambridge CB2 0QQ, United Kingdom; Kuopio Musculoskeletal Research Unit (KMRU), Institute of Clinical Medicine, School of Medicine, University of Eastern Finland (UEF), P.O. Box 1627 FI-70211, Kuopio, Finland

**Keywords:** hip fracture, risk factors, long-term study, aging, osteoporosis, fracture prevention, clinical care

## Abstract

Hip fractures in the aging population are a substantial burden for health care. This study investigates whether risk factors presented in midlife could predict long-term hip fracture risk in the coming decades. The study cohort consisted of 11 169 Finnish women who completed a health survey in 1994, establishing the baseline risk factors. These included all FRAX variables except FN BMD. The national registry data was used to identify hip fractures and deaths. The follow-up baseline was set at the age of 55 and was terminated by hip fracture, death, or the end of the follow-up period, whichever occurred first. The follow-up age ranged from 55 to 90 yr, with a mean follow-up time of 29.5 yr (range 0.1-35.0 yr). Long-term risk estimates were performed for each risk factor individually over a cumulative time of 1-yr intervals, using Cox proportional hazard regression models. In total, 801 hip fractures were observed during the 35 yr. Several midlife risk factors appear to sustain their relevance later in life, predicting long-term hip fracture risk in women. The significant risk factors included (hazard ratio minimum to maximum) previous fracture (HR 1.5-4.4), parental hip fracture (HR 1.3-2.8), regular smoking (HR 1.8-4.1), glucocorticoid use (HR 1.7-5.3), rheumatoid arthritis (HR 2.2-4.8), poor self-reported health (HR 1.0-4.3), and polypharmacy (HR 1.1-2.7). The findings suggest that several risk factors identified in midlife demonstrate persistent associations with the long-term risk of hip fractures. Early detection and prevention of these factors may help to reduce the risk in later life.

## Graphical Abstract

**Graphical Abstract:**
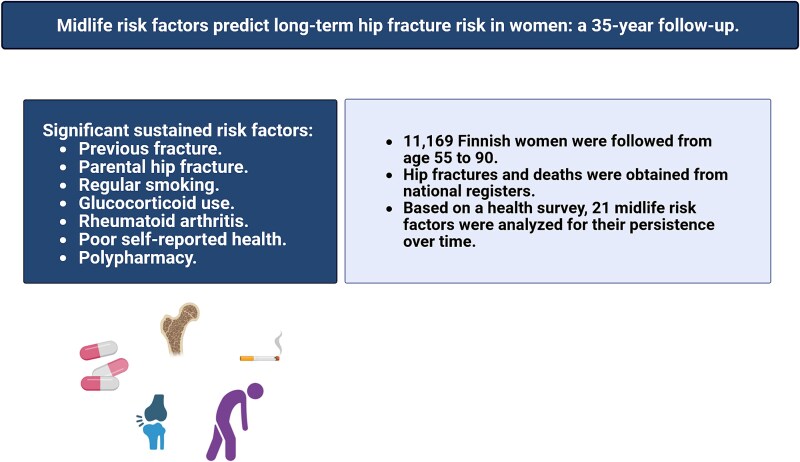

## Introduction

Hip fractures, typically defined as fractures of the proximal femur,^1^ constitute the majority of worldwide fracture-associated public health care expenditure. A fragility fracture report in 2017 estimated that in the largest 5 countries of the European Union plus Sweden (EU6), all fracture-related costs amounted to €37.5 billion, while 19.6% of the fractures were at the hip, accounting for 57% of the total fracture-related costs.^2^^,^^3^ Hip fracture is also associated with a significant impact on the quality of life, long-term physical function, and mortality in the following years.^4–7^

Commonly, hip fracture risk consists of a combination of factors associated with osteoporosis and falls.^8^^,^^9^ As for nonmodifiable risk factors, age and female sex are most strongly associated with hip fractures. In addition, genetic predisposition, family history, and previous fractures also greatly predispose one to hip fracture risk. Among modifiable risk factors, low physical activity and malnutrition are 2 major factors.^2^ Some medications for chronic illnesses, such as glucocorticoids, diuretics, proton pump inhibitors (PPIs), and selective serotonin reuptake inhibitors (SSRIs), may further predispose one to hip fracture.^10^ Further, being underweight is a significant risk factor for osteoporosis and hip fracture. However, body mass index appears to have an age-dependent association with hip fracture. A relationship between obesity and early hip fracture risk has been recognized, while weight fluctuation has been suggested to increase the overall risk of hip fractures.^11^^,^^12^

Hip fracture incidence increases exponentially with age. The median age is around 80, with most patients being over 65.^3^^,^^9^ As such, the number of studies investigating established hip fracture risk factors in midlife and their long-term contribution to the risk is sparse. Even less is known about how these midlife factors maintain their predictive value over the coming decades. Among hip fractures, there is an increased mortality and reduced daily function in older patients.^13^^,^^14^ Smoking, alcohol abuse, poor self-rated health, high or low body mass index, previous fracture, diabetes, and cardiovascular risk factors have been shown to contribute to early hip fractures.^11^^,^^15–17^

Women have a higher incidence of hip fractures and are more commonly studied. In Finland in 2018, women had a 20% higher hip fracture risk compared to men. However, this disparity has significantly diminished in the last 5 decades (relative risk of 1.7 in 1972).^18^ On the contrary, relative risk is higher in younger men compared to younger women, but significantly increased in older women compared to older men.^18^ The behavioral factors behind the risk between genders at different ages are not well-established in the literature. However, in younger men, hip fractures are considered to occur mostly due to high-energy trauma.^18^

This study aimed to assess the association between midlife risk factors and hip fracture risk with their respective change over time in postmenopausal women using FRAX risk factors^19^ and additional health factors acquired from the study survey. Visualizing the interactions between advancing age and the hazard ratios (HRs) associated with a well-established binary (yes/no) risk factor for fracture gives a unique insight into how the factors acquired at midlife persist.

## Materials and methods

### Patients and study design

This prospective cohort study is based on the ongoing Kuopio Osteoporosis Risk Factor and Prevention (OSTPRE) study cohort,^20^ which includes 14 220 women born between 1932 and 1941. It was established in 1989, including the entire age cohort of the women residing in the Kuopio Province, Eastern Finland. OSTPRE utilizes health surveys mailed in 5-yr intervals between 1989 and 2019.

For this study, we used the OSTPRE 1994 (fifth year) health survey as the data source for the analyses. It was sent to 13 864 women, with 12 184 responding (response rate 88%). Out of these, 1015 women (8.3%) were excluded due to missing data on risk factors (*n* = 1002), or due to hip fracture before baseline (*n* = 13). Thus, the final study sample was 11 169 women. For the survival analyses, the follow-up timeline was uniformly anchored at age 55 for all participants. Follow-up was terminated by the first incident, hip fracture, death, or reaching age 90, giving a maximum possible follow-up time of 35 yr.

For women who were younger than 55 at the time of the survey, follow-up counting was delayed until they reached age 55. For women older than 55 in the survey, their follow-up was calculated retrospectively to the standardized age of 55.

Overall, the survey included 52 health-related items, covering factors such as parental hip fracture, history of falls and fractures, menopausal status, lifestyle factors (eg, smoking, alcohol, diet, physical activity), comorbidities, medication use, surgeries, and self-reported health status. The recipient responses have previously been shown to be reliable, for example, with self-reported height and weight.^11^ The questions of interest utilized in the survey are included as supplementary material.

Risk factors were divided into FRAX-based variables^19^ and additional factors. The available FRAX variables included *age, sex, weight, height, previous fracture, parental hip fracture, current smoking, glucocorticoid use, rheumatoid arthritis,* and *alcohol use ≥ 3 units/d*. Secondary osteoporosis was defined per FRAX as the presence of type I diabetes, osteogenesis imperfecta, untreated hyperthyroidism, hypogonadism or early menopause (<45 yr), chronic malnutrition or malabsorption, chronic liver disease. BMD and calculated 10-yr FRAX risk scores were not included in the models.

Additional risk factors from the survey included *poor self-reported health* (categorized as good, mediocre, or poor); *polypharmacy* (defined as ≥5 daily medications); *hormone (estrogen) replacement therapy; regular recreational exercise; one or more falls in the previous 12 mo.; history of ovariectomy; participation in sports at ages 11-17; smoking at ages 11-17; daily milk or coffee consumption; physical workload; use of calcium or vitamin D supplements*; and *any non-gynecologic surgery.*

BMI was calculated using self-reported weight and height and classified using WHO categories: underweight (<18.5 kg/m^2^), normal weight (18.5-24.99), overweight (25-29.99), and obese (≥30.0).

Risk factors were assessed at the time of the 1994 survey, with the women’s age range of 53 to 62 yr. For consistency, all variables were fixed to cover the baseline age of 55 for modeling. This approach allowed uniform time-to-event comparisons while focusing the study objective on exposures measured in midlife (55 yr). Missing responses to individual risk factor questions were excluded from analyses.

The primary outcome was the first incident of hip fracture. Periprosthetic, pathological, and second hip fractures were excluded. Follow-up ended at the time of the first hip fracture, death, or the end of follow-up in 2021, whichever came first.

### Data sources

Hip fracture data was obtained from the Finnish national health registers, including the Hospital Discharge Register and Care Register for Health Care, maintained by the Finnish Institute for Health and Welfare.^21^ The register data is known to identify virtually all hip fractures treated in hospitals.^22^ Dates of death were obtained from the Population Registry. Register linkages included hip fractures and dates of death until the year 2021. Data linkage was possible through personal identity codes.

### Statistical analyses

Cox proportional hazards regression was used to estimate HRs for hip fracture risk. The proportional hazards assumption was tested using covariate*log(time) interaction terms in SPSS and visually inspected via Kaplan–Meier survival curves. No assumption violations were observed. The proportionality tests are included as supplementary material.

To examine how the predictive value of midlife risk factors changed as the cohort aged, we fitted separate Cox proportional hazards models for progressively expanding follow-up windows. The first model evaluated the risk of hip fracture over a 5-yr period from the established baseline (age 55 to 60). Subsequent models extended the upper age limit of the follow-up period in 1-yr increments (eg, age 55 to 61, 55 to 62) up to age 90. Hazard ratios with 95% CIs were plotted for each risk factor to show how their effect varied over the 35-yr period.

We preferred this descriptive approach over a conventional time-varying or interaction-based Cox model, as our primary clinical objective was to evaluate the long-term prognostic value of a single, fixed midlife assessment. While it does not constitute a formal time-dependent covariate analysis, this repeated baseline modeling allows us to illustrate how the predictive utility of midlife risk factors evolves over subsequent decades. Mortality was treated as a censoring event.

The analyses were performed using IBM SPSS Statistics for Windows, version 27 (IBM Corp.). The long-term risk factor estimates were graphed using Microsoft Excel for Windows, version 2412 (Microsoft).

## Results

### Characteristics of the study population

The study population consisted of 11 169 women. In total, 801 hip fractures with a mean age of 77.5 were observed during the 35-yr follow-up. All-cause mortality during the follow-up was 39.1% (*n* = 4362). For the baseline characteristics (Table 1), risk factor analysis between ages 55 and 90 was performed. Overall, the statistically significant midlife risk factors for hip fracture included being underweight, previous fracture, parental hip fracture, regular smoking, glucocorticoid use, rheumatoid arthritis, alcohol use of 3 units or more per day, poor self-reported health, and polypharmacy. Protective factors included hormone replacement therapy (estrogen) and regular recreational exercise.

**Table 1:** Baseline characteristics of the study population, with hazard ratios (HRs) for all risk factors between ages 55 and 90, using the age-adjusted Cox proportional hazard model with their respective HR (95% CI) and *p* values.

**Baseline characteristic**	***n* (%) unless otherwise specified**	**Age-adjusted HR (95% CI) for hip fracture**	***p***
**Mean age at baseline**	55.0		
**Mean follow-up time (range), years**	29.5 (0.1-35.0)		
**Mean age at hip fracture (range), years**	77.5 (55.5-89.8)		
**Mean BMI, kg/m** ^**2**^ **(range)**	27.2 (14.3-61.0)		
**Underweight (BMI < 18.5)**	46 (0.4%)	3.20 (1.43-7.14)	.005^a^
**Normal weight (BMI 18.5-24.99)**	3876 (34.7%)	1.03 (0.89-1.19)	.688
**Overweight (BMI 25-29.99)**	4605 (41.2%)	0.90 (0.78-1.04)	.149
**Obese (BMI *>* 30)**	2598 (23.3%)	1.00 (0.85-1.18)	.988
**Previous fracture (Yes) (any)**	989 (8.9%)	1.51 (1.22-1.88)	<.001^a^
**Parental hip fracture (Yes)**	923 (9.2%)	1.29 (1.03-1.63)	.028^a^
**Regular smoking (Yes)**	1088 (9.7%)	1.81 (1.48-2.22)	<.001^a^
**Glucocorticoid use (Yes)**	512 (4.6%)	1.72 (1.29-2.28)	<.001^a^
**Rheumatoid arthritis (Yes)**	379 (3.4%)	2.23 (1.66-3.01)	<.001^a^
**Secondary osteoporosis (Yes)**	1762 (15.8%)	1.09 (0.91-1.32)	.347
**Alcohol use of 3 or more units per day (Yes)**	17 (0.2%)	7.44 (3.08-17.97)	<.001^a^
**Poor self-reported health (Yes)**	1478 (13.2%)	1.45 (1.20-1.75)	<.001^a^
**Polypharmacy (Yes)**	702 (6.3%)	1.79 (1.39-2.30)	<.001^a^
**Hormone replacement therapy (Yes)**	4619 (41.4%)	0.72 (0.62-0.83)	<.001^a^
**Regular recreational exercise (Yes)**	5342 (47.8%)	0.85 (0.74-0.98)	.028^a^
**One or more falls during the last 12 mo (Yes)**	3634 (32.5%)	1.01 (0.87-1.18)	.898
**Ovariectomy (Yes)**	1011 (9.1%)	0.93 (0.72-1.19)	.558
**Sport at 11-17 yr of age (Yes)**	5010 (44.9%)	1.04 (0.90-1.20)	.616
**Smoking at 11-17 yr of age (Yes)**	245 (2.2%)	1.37 (0.88-2.14)	.164
**Daily milk product use (Yes)**	10 247 (91.7%)	1.05 (0.79-1.38)	.747
**Daily coffee use (Yes)**	10 553 (94.5%)	0.89 (0.65-1.22)	.461
**Considers their work laborious (Yes)**	2948 (26.4%)	1.08 (0.91-1.26)	.384
**Use of calcium or vitamin D supplements (Yes)**	3888 (34.8%)	0.89 (0.77-1.03)	.127
**Any non-gynecologic surgery (Yes)**	6534 (58.5%)	0.92 (0.79-1.06)	.250

a *p* values under .05 are considered significant.

### Estimating long-term hip fracture risk

**Figure 1:**
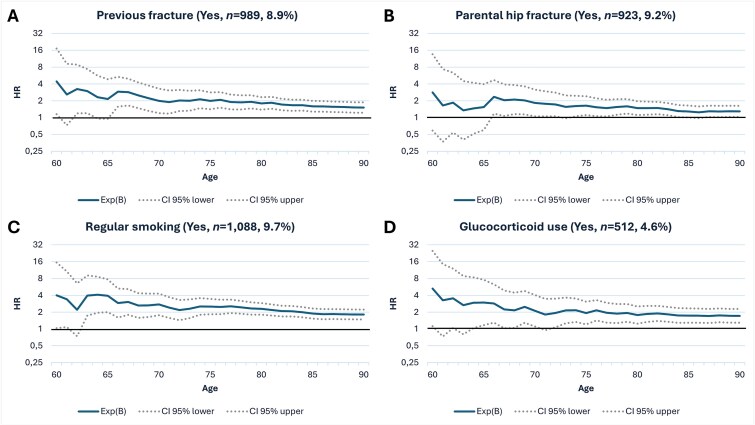
Hazard ratios for variables affecting long-term hip fracture risk, including previous fracture, parental hip fracture, regular smoking, and glucocorticoid use. Cox regression analyses with a hazard ratio (HR) y-scale in blue and 95% CI in a dotted continuous line.

In terms of fracture history, the women with a previous fracture had an HR of 1.5-4.4 (Figure 1A) and the women who had a parent with a fractured hip showed an elevated risk with an HR of 1.3-2.8 (Figure 1B). In both cases, HR was at its highest at the age of 60, while the associations started to diminish after the age of 70.

For lifestyle variables, regular smoking showed a significant long-term risk with an HR of 1.8-4.1 (Figure 1C), peaking at age 64 and diminishing over time. However, the effect persisted into later years, dropping below 2.0 after the age of 84. In addition, excessive alcohol use (of 3 or more units per day) had a high HR of 7.4, but with a small sample size (*n* = 17), the results remain inconclusive (Table 1).

**Figure 2:**
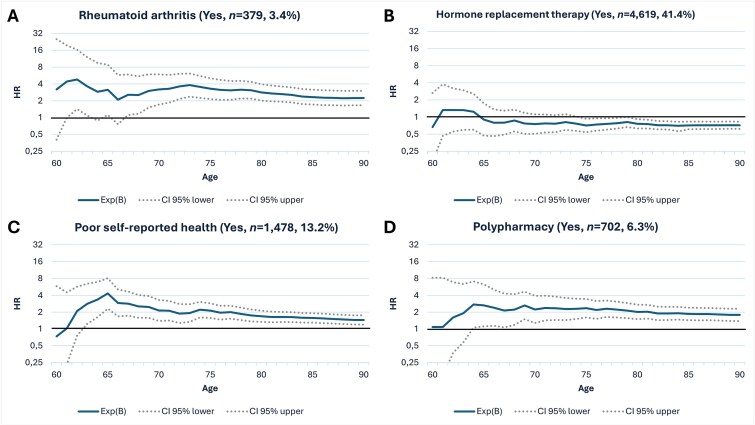
Hazard ratios (HRs) for variables affecting long-term hip fracture risk, including rheumatoid arthritis, hormone replacement therapy, poor self-reported health, and polypharmacy. Cox regression analyses with an HR y-scale in blue and 95% CI in dotted continuous line.

Several medications and morbidities, such as glucocorticoid use (Figure 1D) and rheumatoid arthritis (Figure 2A), were shown to be major long-term risk factors, with both peaking around an HR of 5.0 (5.3 and 4.8, respectively) at 60-62 yr of age and diminishing over time. They both reached their lowest HRs at age 90 (1.7 and 2.2, respectively). In turn, hormone replacement therapy (Figure 2B) was considered a protective factor only after the age of 65. Poor self-reported health demonstrated a significant long-term risk, with its highest HR (4.3) observed at age 65. The effect seems to diminish over time, falling below 2.1 after age 75 (Figure 2C). Overall, midlife polypharmacy with 5 or more medications was a notable long-term risk factor with an HR of 1.1-2.7, peaking at age 64. The risk seems to stabilize around an HR of 2.0 throughout the women’s late adulthood (Figure 2D).

Secondary osteoporosis was not associated with hip fractures in this cohort, as its HR appeared around 1.0 throughout the follow-up. Considering BMI, being underweight was a significant risk factor for hip fracture, with an HR of 2.9-8.0. The other BMI categories did not show a statistically significant long-term trend between the normal, overweight, or obese women.

## Discussion

This study demonstrated that many previously recognized risk factors for hip fracture, measured in midlife, show persistent associations with fracture risk into later life. The most important long-term risk factors were previous fracture, regular smoking, glucocorticoid use, rheumatoid arthritis, and poor self-reported health. On the contrary, hormone replacement therapy and regular physical exercise at midlife were found to be lifelong protective factors for hip fracture risk. However, it should be noted that, as hip fractures accumulate, most of the midlife risk factors only start to show statistically reasonable estimates at around 65 yr of age and should be interpreted accordingly.

In most cases, the HRs for the risk factors appear to be highest among women in their 60s, and the risks gradually diminish after the women reach their 70s or 80s. Expectedly, as these factors were reported at midlife, numerous other factors modify and contribute to their change over time, explaining the HR fluctuations. As aging itself is a significant risk, overlapping factors such as deteriorating bone mass and frailty additionally increase the susceptibility to fractures and falls. However, in this study, the analyses were age-adjusted, with the follow-up starting at age 55 to control the effect of aging alone.

Several contributing factors affecting the overall results of this observational long-term study should be considered. Since the risk factors were recorded at age 55, the HR reflects the changing impact of these factors on fracture risk over time. The peak HR at age 55 indicates that individuals with any risk factor of interest are at their highest relative risk for fractures at their current age. As individuals age, the weight of these factors is expected to change due to various reasons, such as changes in health status, lifestyle adaptations, or the natural progression of aging. For example, women who smoke may experience a more rapid health decline, exposing themselves to fractures, while others can modify their behavior more favorably (eg, quitting smoking) or receive medical interventions that reduce their risk. In addition, as the follow-up progresses, survivor bias will also become a factor. Individuals who are at higher risk (eg, those with a previous fractures or who smoke) may experience fractures and increased mortality. This can lead to a lower observed risk in older age, as the remaining cohort may consist of individuals who are healthier, even though the absolute risk remains high. Overall, the cumulative effects of aging and the interaction with the midlife risk factors will lead to increasingly more complex relationships over time. For instance, biological aging is likely to exacerbate some of the existing risk factors, such as smoking or previous fractures, even further. Similarly, several factors may diminish due to the same reasons mentioned above.

The relationship between time-dependent BMI and the risk of hip fracture, previously reported in this cohort, showed that initial midlife obesity is associated with early hip fracture risk before 70 yr of age.^11^ However, the midlife BMI alone did not sustain a clear lifelong difference between the categories of normal weight, overweight, and obese into old age. Being underweight maintained the highest overall risk with a 2.9- to 8.0-fold increase throughout the follow-up period. However, it should be noted that only a very small proportion of women (*n* = 46, 0.4%) were considered underweight (BMI <18.5), affecting the reliability of these findings.

Smoking and alcohol abuse are known to be associated with hip fracture risk. The BMD loss is greater compared to non-smoking women, especially after 60.^23^ Heavy smokers might have higher bone loss and deteriorated bone quality, as smoking has a dose-dependent risk and the effect appears to be cumulative.^23^^,^^24^ In our study, midlife smoking introduced a 2- to 4-fold increase in HR, affecting women under 70 the most. The association between alcohol consumption and hip fracture risk appears to be non-linear. Heavy consumption has been associated with elevated hip fracture risk, while light consumption has been correlated with increased FN BMD.^25^^,^^26^ Here, higher alcohol use manifested as the strongest risk factor with a HR of 7.4, but without a clear long-term trend. However, only an extremely small proportion of women (*n* = 17, 0.2%) reported using alcohol 3 units or more per day. Thus, the results remain inconclusive.

Poor self-reported health has been linked to an increased risk of hip fracture in both midlife and late adulthood among women. A Swedish study of participants aged 48 to 68 recorded 135 hip fractures in women and identified poor self-rated health as one of the study’s strongest risk factors, with a risk ratio of 1.74.^15^ Another Swedish study involving 350 women aged 69 to 79 found a significantly higher risk of hip fracture in those who rated their health as “low” (HR 3.17) or “intermediate” (HR 2.75).^27^ In our cohort, poor self-reported health at midlife was associated with a 2- to 4.3-fold increased risk before age 70, with the gradually decreasing impact over time.

The independent impact of polypharmacy on hip fracture risk alone is not well-known. Patients with multiple medications typically have multiple conditions, which themselves may predispose to the risk of hip fracture more or less than the pharmacological side effects alone. When analyzing individual medications, hip fracture risk is shown to increase with loop diuretics, SSRIs, PPIs, and chemotherapeutic agents^28^^,^^29^ while the link between psychiatric polypharmacy and hip fractures remains inconclusive.^30^^,^^31^ In this cohort, polypharmacy appeared to be a steady risk factor with a HR of 1.6 to 2.7 throughout the aging women’s lifespan. Prolonged glucocorticoid use has been suggested to decrease BMD and increase the relative risk of hip fracture, even with a low, under 5 mg per day dose.^32–35^ Here, glucocorticoid use showed a HR of up to 5.3 around 60 yr of age, gradually lowering to around 2.0 between the ages of 68 and 80.

Comorbidities are widely studied as hip fracture risk factors. Rheumatoid arthritis decreases BMD density due to inflammation and glucocorticoid use.^36^ In addition, joint pain and limited range of motion may also contribute to falls and elevated fracture risk in these women. Thus, patients with the disease are shown to have a higher incidence of early hip fractures.^36–38^ In our analysis, rheumatoid arthritis showed a somewhat stable, 2- to 4-fold increase in HR, peaking at 4.8 around the age of 60. In secondary osteoporosis, BMD and the microarchitectural structure are compromised due to an underlying disease or medication, leading to an increase in susceptibility to fracture.^39–41^ Studies investigating the secondary condition as an independent hip fracture risk are sparse, with 1 study concluding that the estimated risk for hip fracture was higher in women than men (9.1% vs 4.4%).^40^ In our study, secondary osteoporosis did not increase the long-term risk of hip fracture.

Previous fracture and fall history predict future falls.^42^ Patients with previous fractures have also been noted to have decreased BMD and bone quality. In the literature, previous fracture history has corresponded to a risk ratio of around 1.7 when considering all ages.^43^^,^^44^ In our cohort, women with previous fracture history had the highest HR of 4.4 at 60 yr of age, declining steadily to around 2 at 70 yr of age.

A parental history of hip fracture is a strong, BMD-independent predictor of future fracture risk. In 2025, a large-scale meta-analysis updating the FRAX tool confirmed this association, reporting an HR of 1.37 for future hip fractures across international cohorts.^45^

In addition, earlier parental fracture for the parent has been associated with a greater risk of a major osteoporotic fracture in offspring.^46^ Here, parental hip fracture had a peak HR of 2.8 at the age of 60.

During menopause, decreased estrogen levels result in decreased BMD. Still, routine therapy is not typically recommended due to adverse conditions.^47^ In this study, the individual indication, or dose, for hormone therapy was not included. Yet, the reported use at midlife appeared to have a protective effect against hip fracture later in life (HR below 0.90 after 65).

Regular exercise is one of the most effective individual methods to reduce the rate of falls and fall injuries in the aging population.^48–50^ Self-reported physical exercise was not specified according to type or duration in this study, but it seems to have a significant protective effect on hip fracture risk (HR 0.85).

This study included all commonly used FRAX risk factors, along with several additional factors of interest from midlife, to assess their contribution to long-term fracture risk in aging women. All FRAX variables, except for secondary osteoporosis, were shown to contribute and persist as long-term risk factors into old age. However, while they appear to sustain their relevance, several of the risk factors remain modifiable and are prone to inevitable changes over time. This supports the current policy of using 10-yr fracture risk estimations in clinical use.

The study has certain limitations. Although we examined associations over a long follow-up period, we observed no violation of the proportional hazards assumption, supporting the use of Cox regression models. However, as we examined changes in HRs over time, this should not be interpreted as a formal time-dependent covariate analysis, but an exploratory repeated baseline modeling approach to observe age-related risk patterns. Given the modeling across multiple risk factors and time intervals, different confounding factors cannot be excluded. As mortality was treated as a censoring event, further analysis of competing risk models incorporating mortality could yield further estimates. Although this was not in the clinical scope of this study, the approach should be explored in future work. Additionally, secular societal changes during the long follow-up, such as evolving medical care and public health policies, could influence exposure and outcome but were not analyzed and may introduce some bias. With these considerations, the findings should be contextualized accordingly. Future investigations should utilize competing risk models to assess how competing mortality influences these age-related patterns.

In conclusion, we found that risk factors identified in midlife, including previous fracture, parental hip fracture, regular smoking, glucocorticoid use, rheumatoid arthritis, poor self-reported health, and polypharmacy, persist and significantly increase the lifelong risk of hip fractures in aging women. Early detection and preventative strategies started in early postmenopausal years could mitigate the risk of hip fracture in later life.

## Supplementary Material

Appendix_1_OSTPRE_1994_survey_ziag083

Appendix_2_Additional_figures_ziag083

Appendix_3_Cox_proportional_hazard_assumption_tests_ziag083

## Data Availability

The data that support the findings of this study are available on reasonable request from the authors for academic studies. The data is not publicly available due to privacy or ethical restrictions.
