# Supplementary material for: Midlife risk factors predict long-term hip fracture risk in women: a 35-yr follow-up
Source: JBMR Plus. 2026 May 8;10(6):ziag083. doi: 10.1093/jbmrpl/ziag083 (PMC13198794; doi:10.1093/jbmrpl/ziag083)
Supplement: Appendix_1_OSTPRE_1994_survey_ziag083 [file appendix_1_ostpre_1994_survey_ziag083.pdf]

Relevant questions used from the OSTPRE 1994 survey. Original translation by Risto Honkanen in 1995.

Has your MOTHER or FATHER fractured her/his HIP?

How many times have you YOURSELF FALLEN ON THE SAME LEVEL DURING THE PREVIOUS YEAR (12 MONTHS) including falls without injury?

Have you had BONE FRACTURES DIAGNOSED BY A PHYSICIAN SINCE 1.1.1989?

Have your both ovaries been removed?

Have you used female hormone therapy as tablets, gel or skin plaster since the 1st of June 1989?

Did you participate in sports on your own initiative in addition to school physical education WHEN 11-17 YEAR OLD?

Were you smoking WHEN 11-17 YEAR OLD?

Are you smoking at present?

Your weight at present?

Your height at present?

How many deciliters of milk products (f.e. milk, sour milk, yoghurt, cheese) are you using daily AT PRESENT?

How many cups of coffee do you drink on an average daily AT PRESENT?

What is the amount of alcohol beverages you are using?

Have you had regular LEISURE physical activity during the previous year?

How physically strenuous is/was your work?

Is your health status compared to that of other same-aged POOR?

Has a physician diagnosed following diseases in you?

1. Sight impairment affecting moving
2. Hypertension requiring drug treatment
3. Coronary heart disease (infarct, angina)
4. Other heart disease (f.e. h. failure)
5. Apoplexy, cerebral hemorhage or infarct
6. Diabetes treated with insulin
7. Diabetes requiring tablet treatment
8. Hyperthyroidism
9. Hypothyroidism
10. Coeliacia
11. Stomach resection
12. Chronic kidney disease
13. Chronic liver disease
14. Arthrosis affecting moving
15. Rheumatoid arthritis
16. Chronic back pain
17. Epilepsy
18. Pulmonary asthma
19. Other chronic pulmonary disease
20. Alcoholism
21. Chronic mental disease
22. Cancer, specify
23. Other chronic or severe diseases, specify

24. None of the above-mentioned or other chronic or severe diseases

Are you using DRUGS PRESCRIBED BY A PHYSICIAN at present?

Have you ever used CORTISONE as tablets (f.e. Prednisolon, Medrol) or as bronchial or nasal inhalation (f.e. Pulmicort, Becotide) for at least one week?

Are you using daily CALCIUM OR VITAMIN D containing drugs or natural products?

Have you underwent other than gynaecological operations?
