# Supplementary material for: Midlife risk factors predict long-term hip fracture risk in women: a 35-yr follow-up
Source: JBMR Plus. 2026 May 8;10(6):ziag083. doi: 10.1093/jbmrpl/ziag083 (PMC13198794; doi:10.1093/jbmrpl/ziag083)
Supplement: Appendix_2_Additional_figures_ziag083 [file appendix_2_additional_figures_ziag083.pdf]

Supplementary Fig. 1. Hazard ratios for variables affecting long-term hip fracture risk, including normal weight (A), overweight (B), obese (C), underweight (D), and secondary osteoporosis (E). Cox regression analyses with hazard ratio (HR) y-scale and 95% confidence interval.

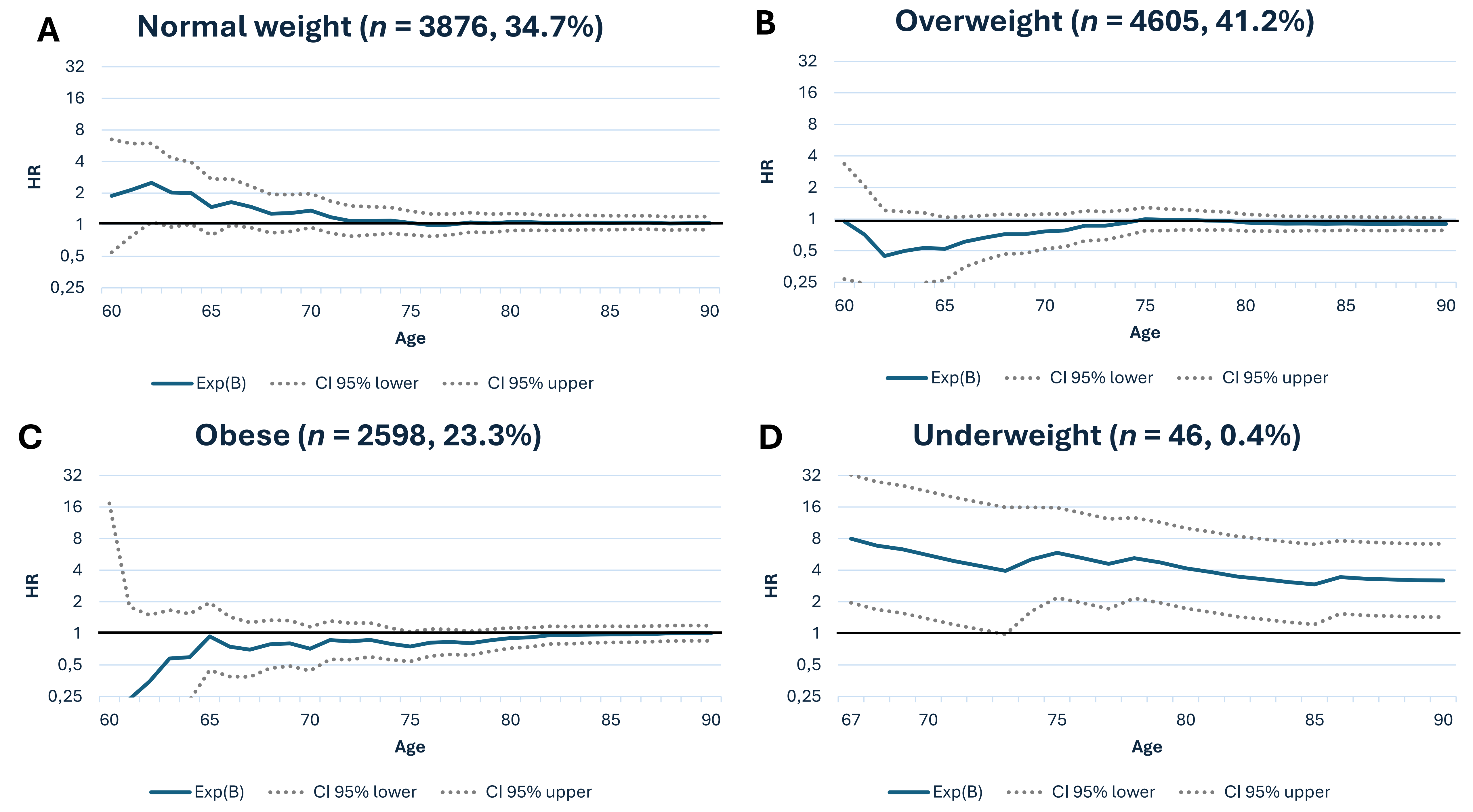

A
Normal weight (n = 3876, 34.7%)
B
Overweight (n = 4605, 41.2%)
C
Obese (n = 2598, 23.3%)
D
Underweight (n = 46, 0.4%)
HR
32
16
8
4
2
1
0,5
0,25
60
65
70
75
80
85
90
67
Age
Exp(B)
CI 95% lower
CI 95% upper

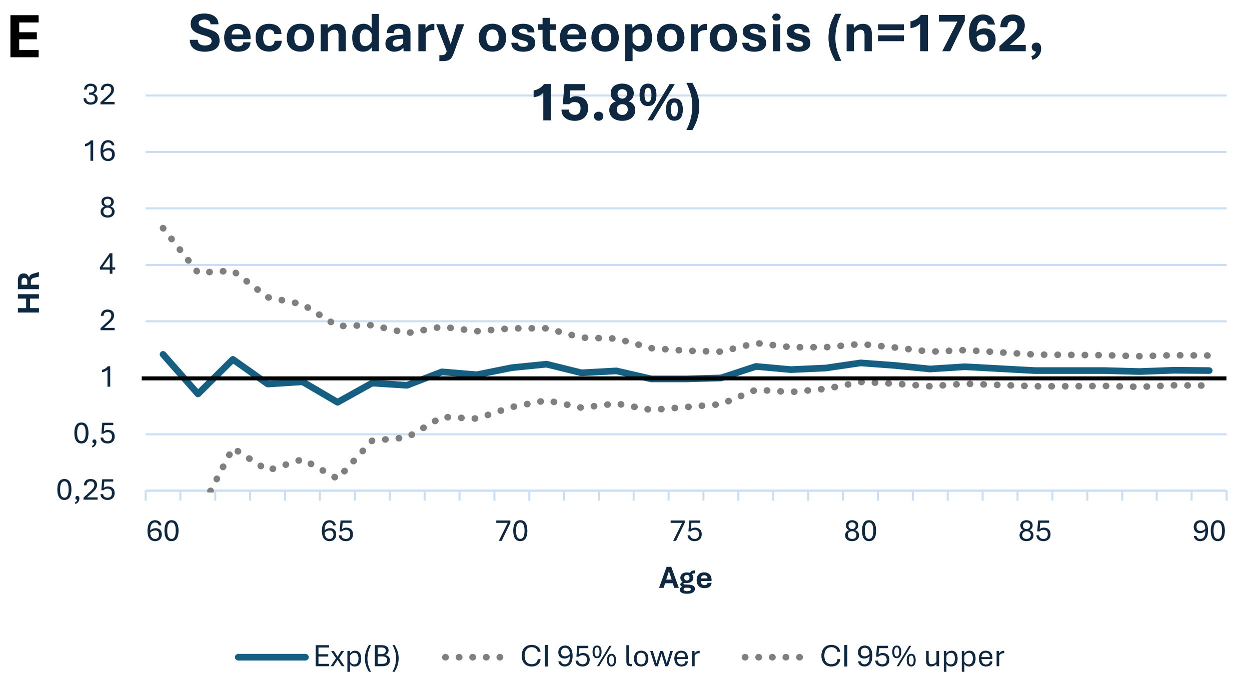

E
Secondary osteoporosis (n=1762, 15.8%)
HR
32
16
8
4
2
1
0,5
0,25
60
65
70
75
80
85
90
Age
Exp(B)
CI 95% lower
CI 95% upper
