# Supplementary material for: Midlife risk factors predict long-term hip fracture risk in women: a 35-yr follow-up
Source: JBMR Plus. 2026 May 8;10(6):ziag083. doi: 10.1093/jbmrpl/ziag083 (PMC13198794; doi:10.1093/jbmrpl/ziag083)
Supplement: Appendix_3_Cox_proportional_hazard_assumption_tests_ziag083 [file appendix_3_cox_proportional_hazard_assumption_tests_ziag083.pdf]

The proportional hazards assumption for all variables was tested for proportionality by including covariate*log(time) with a follow-up age from 55 to 90 in SPSS (Supplementary Table 1). No assumption violations were observed (p>0.9 for all variables).

Supplementary Table 1. Proportional hazards assumption for all variables by including covariate*log(time) with a follow-up age from 55 to 90.

**Variable**	**p**
Alcohol use of three or more units per day (Yes)	1.149
BMI Normal weight (BMI 18.5–24.99)	1.059
BMI Obese (BMI >30)	0.906
BMI Overweight (BMI 25–29.99)	1.012
BMI Underweight (BMI <18.5)	1.129
Regular smoking (Yes)	1.006
Hormone replacement therapy (Yes)	1.022
Glucocorticoid use (Yes)	1.031
Parental hip fracture (Yes)	1.066
Polypharmacy (Yes)	0.944
Poor self-reported health (Yes)	0.953
Previous fracture (Yes) (any)	1.034
Rheumatoid arthritis (Yes)	1.000
Secondary osteoporosis (Yes)	0.991
Regular recreational exercise (Yes)	1.022
One or more falls during the last 12 months (Yes)	1.015
Ovariectomy (Yes)	0.959
Sport at 11–17 years of age (Yes)	1.013
Smoking at 11–17 years of age (Yes)	0.936
Daily milk product use (Yes)	0.976
Daily coffee use (Yes)	0.976
Considers their work laborious (Yes)	0.983
Use of calcium or vitamin D supplements (Yes)	0.993
Any non-gynecologic surgery (Yes)	0.948

In addition, Kaplan–Meier curves were visually inspected for potential violations of proportionality, e.g., crossing curves or convergence over time for variables affecting long-term hip fracture risk (Supplementary Fig. 2–9). No violations were observed.

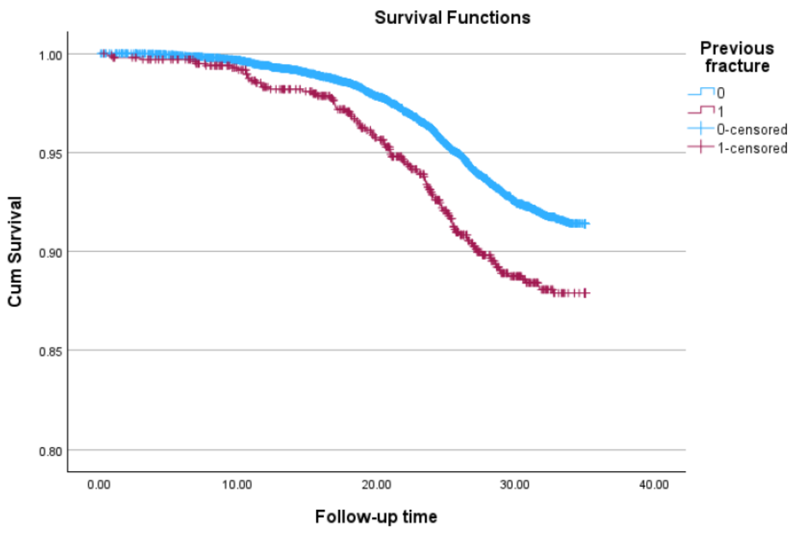

Supplementary Fig. 2. Kaplan–Meier survival curve for previous fracture.

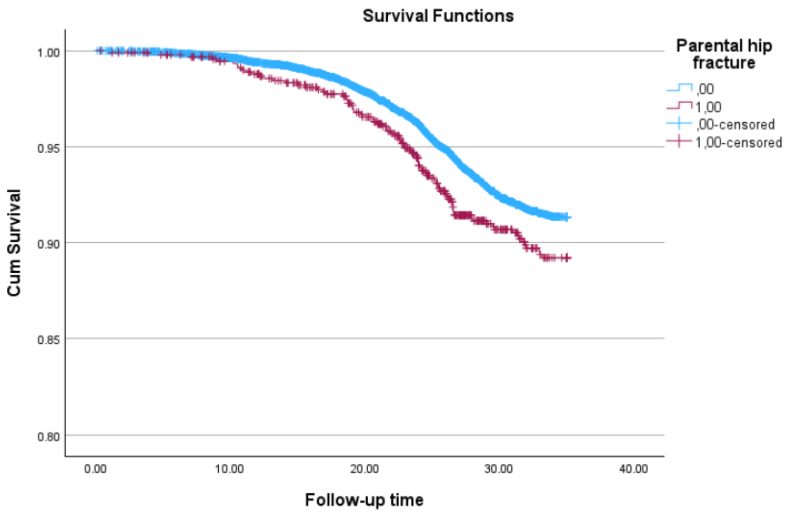

Supplementary Fig. 3. Kaplan–Meier survival curve for parental hip fracture.

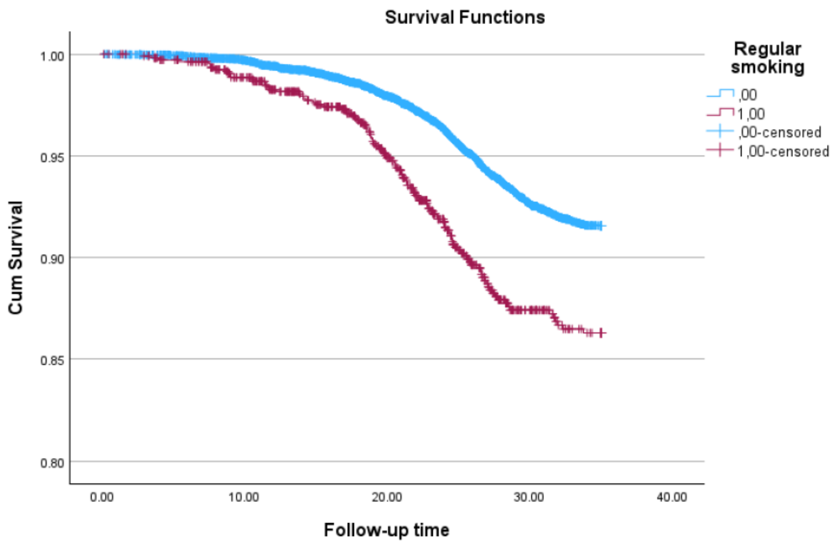

Supplementary Fig. 4. Kaplan–Meier survival curve for regular smoking.

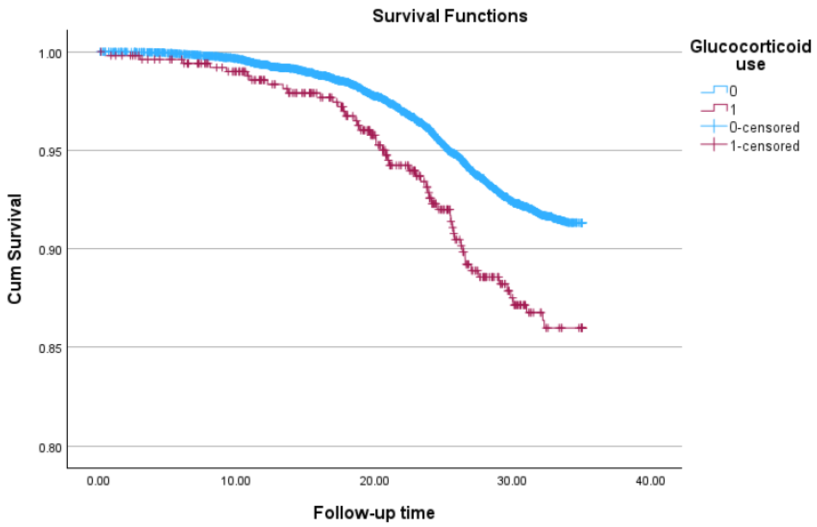

Supplementary Fig. 5. Kaplan–Meier survival curve for glucocorticoid use.

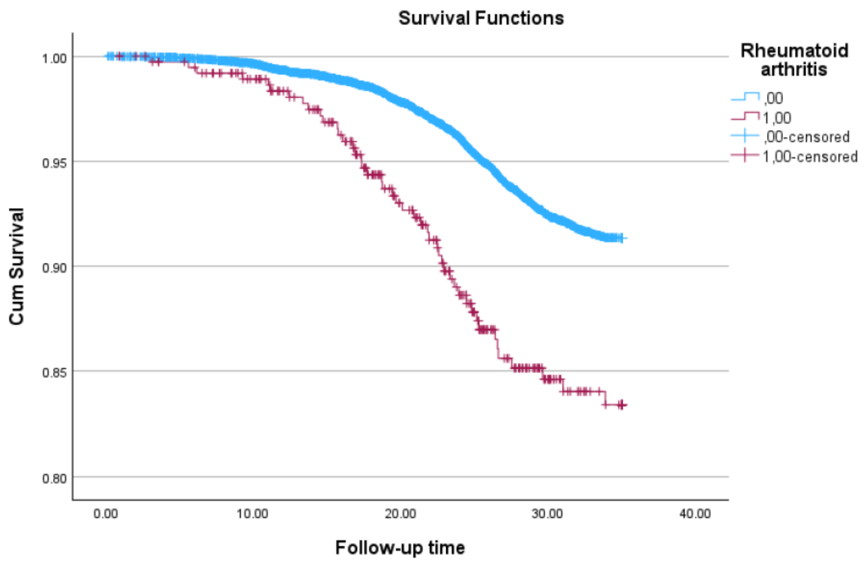

Supplementary Fig. 6. Kaplan–Meier survival curve for rheumatoid arthritis.

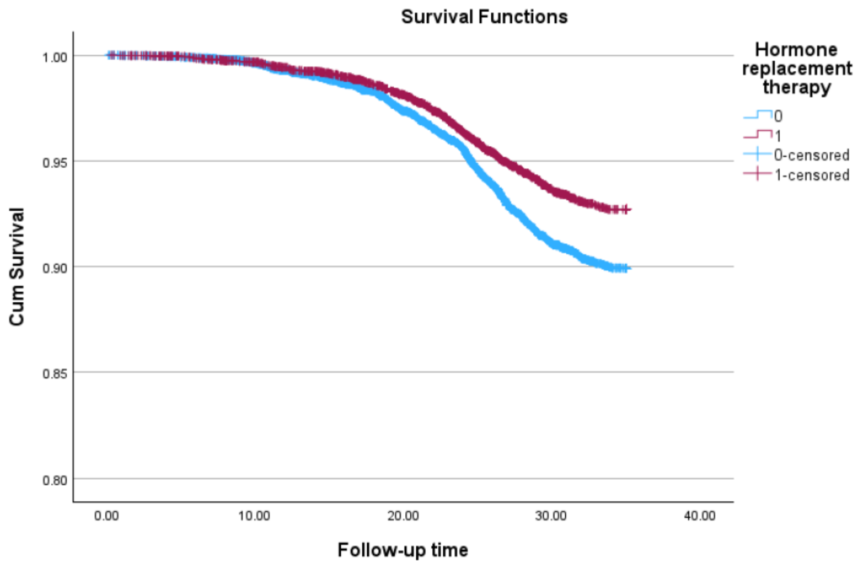

Supplementary Fig. 7. Kaplan–Meier survival curve for hormone replacement therapy.

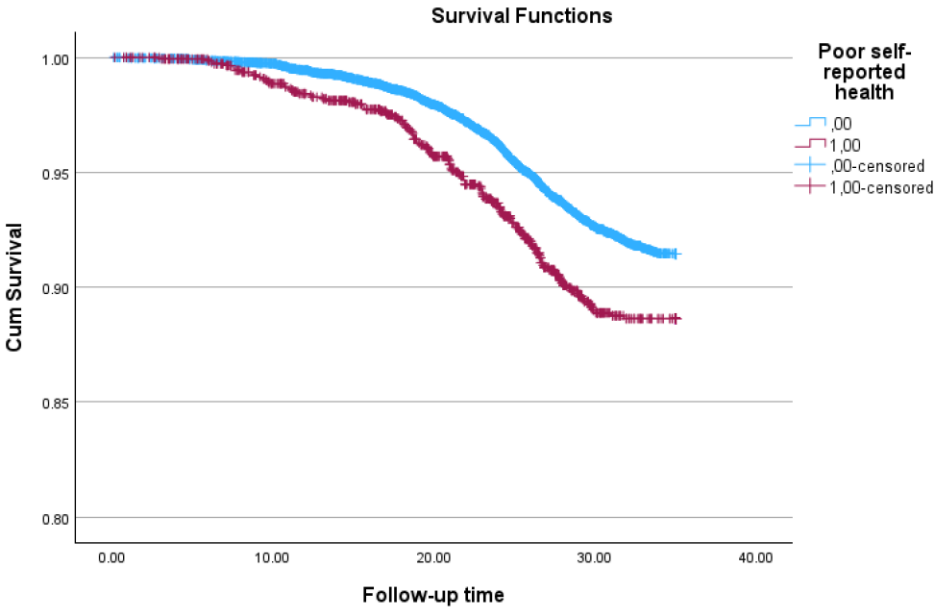

Supplementary Fig. 8. Kaplan–Meier survival curve for poor self-rated health.

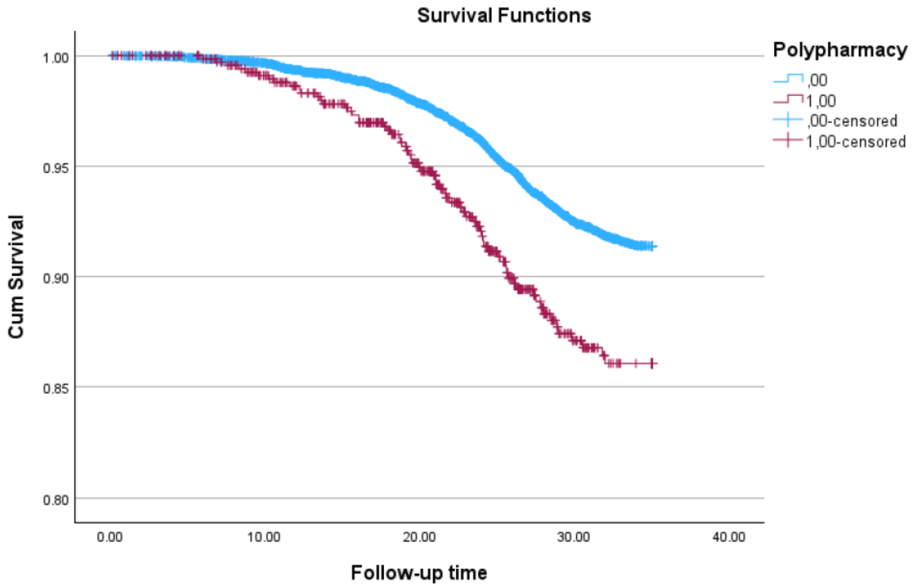

Supplementary Fig. 9. Kaplan–Meier survival curve for polypharmacy.
